# Supplementary material for: Chemical Variability and In Vitro Anti-Inflammatory Activity of Leaf Essential Oil from Ivorian Isolona dewevrei (De Wild. & T. Durand) Engl. & Diels
Source: Molecules. 2021 Oct 15;26(20):6228. doi: 10.3390/molecules26206228 (PMC8539547; doi:10.3390/molecules26206228)
Supplement: Supplementary file 1 [file molecules-26-06228-s001.zip › molecules-1402691-supplementary.pdf]

Supplementary Materials:

Chemical Variability and *in vitro* Anti-inflammatory Activity of Leaf Essential Oil from Ivorian *Isolona dewevrei* (De Wild. & T. Durand) Engl. & Diels

Didjour Albert Kambiré [1], Jean Brice Boti [2], Ahmont Claude Landry Kablan [1], Daouda Ballo [2], Mathieu Paoli [3], Virginie Brunini [3], and Félix Tomi [3,*]

[1] UPR de Chimie Organique, Département de Mathématiques, Physique et Chimie, UFR des Sciences Biologiques, Université Péléforo Gon Coulibaly, BP 1328 Korhogo, Côte d'Ivoire; dakambire@gmail.com (D.A.K.); kablanahmont@yahoo.fr (A.C.L.K.)

[2] Laboratoire de Constitution et Réaction de la Matière, UFR-SSMT, Université Félix Houphouët-Boigny, BP V34 Abidjan, Côte d'Ivoire; jeanbriceboti@hotmail.fr (J.B.B.); daoudaballo526@gmail.com (D.B.)

[3] Laboratoire Sciences Pour l'Environnement, Université de Corse—CNRS, UMR 6134 SPE, Route des Sanguinaires, 20000 Ajaccio, France; paoli_m@univ-corse.fr (M.P.); brunini_v@univ-corse.fr (V.B.)

* Correspondence: tomi_f@univ-corse.fr

Received: Accepted: Published:

Abstract: The chemical variability and the *in vitro* anti-inflammatory activity of the leaf essential oil from Ivorian *Isolona dewevrei* were investigated for the first time. Forty-seven oil samples were analyzed using combination of CC, GC(RI), GC-MS and ^{13}C-NMR, thus leading to the identification of 113 constituents (90.8-98.9%). As the main components varied drastically from sample to sample, the 47 oil compositions were submitted to hierarchical cluster and principal components analyses. Three distinct groups, each dividing into two subgroups were evidenced. Subgroup I-A was dominated by (*Z*)-β-ocimene, β-eudesmol, germacrene D and (*E*)-β-ocimene, while (10βH)-1β,8β-oxido-cadina-4-ene, santalenone, *trans*-α-bergamotene and *trans*-β-bergamotene were the main compounds of Subgroup I-B. The prevalent constituents of Subgroup II-A were germacrene B, (*E*)-β-caryophyllene, (5αH,10βMe)-6,12-oxido-elema-1,3,6,11(12)-tetraene and γ-elemene. Subgroup II-B displayed germacrene B, germacrene D and (*Z*)-β-ocimene as majority compounds. Germacrene D was the most abundant constituent of Group III, followed in Subgroup III-A by (*E*)-β-caryophyllene, (10βH)-1β,8β-oxido-cadina-4-ene, germacrene D-8-one, then in subgroup III-B by (*Z*)-β-ocimene and (*E*)-β-ocimene. The observed qualitative and quantitative chemical variability was probably due to combined factors, mostly phenology and season, then harvest site to a lesser extent. The lipoxygenase inhibition by a leaf oil sample was also evaluated. The oil IC_{50} (0.020±0.005 mg/mL) was slightly higher than the non-competitive lipoxygenase inhibitor NDGA IC_{50} (0.013±0.003 mg/mL), suggesting a significant *in vitro* anti-inflammatory potential.

Keywords: *Isolona dewevrei*; leaf essential oil; chemical variability; *in vitro* anti-inflammatory activity

Table S1: Chemical composition of the 47 leaf essential oil samples from *Isolona dewevrei*.

Table S2: Plant material, essential oil extraction and climate data.

Table S1. Chemical composition of the 47 leaf essential oil samples from *Isolona dewevrei* (Samples 1 to 24).

	Compounds	RIa	RIp	1	2	3	4	5	6	7	8	9	10	11	12	13	14	15	16	17	18	19	20	21	22	23	24	Identification
1	α-Thujene	923	1016	0.3	0.2	0.2	0.6	0.5	0.7	0.9	0.6	0.5	0.5	0.6	0.6	0.5	0.5	0.6	0.5	tr	0.5	0.1	0.1	0.8	0.2	1.5	tr	RI, MS, ^{13}C-NMR
2	α-Pinene	931	1013	0.1	0.1	0.1	0.3	0.4	0.6	0.8	0.5	0.4	0.4	0.4	0.4	0.4	0.4	0.5	0.4	0.1	0.4	0.1	0.1	0.4	0.1	1.3	0.1	RI, MS, ^{13}C-NMR
3	Sabinene	965	1120	0.8	0.6	0.7	2.2	2.0	2.8	3.4	2.6	2.3	1.5	2.1	2.0	1.7	1.8	2.0	1.8	0.6	1.9	0.4	0.4	2.9	0.6	5.0	0.1	RI, MS, ^{13}C-NMR
4	β-Pinene	970	1109	0.1	tr	tr	0.3	0.4	0.6	0.9	0.5	0.5	0.4	0.5	0.4	0.4	0.4	0.5	0.4	0.1	0.4	0.2	0.2	0.3	0.2	1.6	0.1	RI, MS, ^{13}C-NMR
5	Myrcene	981	1158	0.3	0.2	0.3	0.7	0.5	0.5	0.7	0.6	0.6	0.5	0.6	0.7	0.4	0.6	0.4	0.5	0.1	0.6	0.3	0.3	1.0	0.4	1.6	0.3	RI, MS, ^{13}C-NMR
6	α-Phellandrene	997	1162	tr	tr	tr	tr	tr	tr	tr	tr	tr	tr	tr	0.1	tr	0.1	tr	tr	-	tr	tr	tr	0.1	tr	0.1	tr	RI, MS
7	δ-3-Carene	1 005	1146	tr	tr	-	tr	tr	tr	tr	tr	tr	tr	0.1	0.1	tr	0.1	tr	tr	-	0.1	tr	tr	0.1	tr	0.1	tr	RI, MS
8	α-Terpinene	1 009	1178	0.1	tr	0.1	0.2	0.1	0.1	0.2	0.2	0.2	0.1	0.1	0.2	0.1	0.1	0.1	0.1	-	0.1	0.1	0.1	0.3	0.1	0.5	0.1	RI, MS, ^{13}C-NMR
9	*p*-Cymene	1 012	1268	0.7	0.4	0.2	0.5	0.2	0.3	0.5	0.2	0.1	0.5	0.7	0.4	0.6	0.3	1.3	0.5	1.0	0.5	0.1	0.1	0.4	0.1	0.4	tr	RI, MS, ^{13}C-NMR
10	Limonene	1 021	1199	0.5	0.1	0.1	2.0	3.2	4.1	6.3	3.7	3.5	3.1	3.6	3.1	2.9	2.4	3.9	2.9	3.9	3.3	1.1	1.2	2.1	1.2	10.8	1.1	RI, MS, ^{13}C-NMR
11	(*Z*)-β-Ocimene	1 025	1230	1.0	0.8	12.4	9.7	3.4	3.3	3.0	3.7	4.2	4.8	6.2	8.1	5.2	7.8	4.6	6.3	0.1	7.6	4.5	4.7	22.7	5.9	6.7	3.4	RI, MS, ^{13}C-NMR
12	(*E*)-β-Ocimene	1 036	1247	1.2	0.5	1.8	4.1	2.8	2.3	2.2	2.8	2.9	2.7	3.7	5.1	3.9	4.8	3.7	4.2	-	4.6	4.2	4.9	6.2	4.4	4.1	4.5	RI, MS, ^{13}C-NMR
13	γ-Terpinene	1 048	1242	0.2	0.2	0.3	0.4	0.3	0.3	0.4	0.3	0.4	0.2	0.3	0.4	0.3	0.3	0.3	0.3	-	0.4	0.2	0.2	0.6	0.2	1.1	0.2	RI, MS, ^{13}C-NMR
14	Terpinolene	1 078	1279	tr	tr	tr	0.1	0.1	0.1	0.1	0.1	0.1	0.2	0.2	0.1	0.2	0.1	0.3	0.2	0.2	tr	tr	tr	0.2	tr	0.2	tr	RI, MS
15	Linalool	1 083	1543	0.1	0.1	0.1	0.1	1.6	0.6	0.4	0.4	0.1	0.1	0.1	0.2	0.1	0.2	0.5	0.1	0.2	0.2	0.1	0.1	0.2	0.1	0.2	tr	RI, MS, ^{13}C-NMR
16	*allo*-Ocimene	1 117	1370	0.1	0.1	0.4	0.3	0.1	0.1	0.1	0.1	tr	0.1	0.1	0.2	0.1	0.2	0.1	0.2	0.1	0.2	0.1	0.1	0.7	0.2	0.2	0.1	RI, MS, ^{13}C-NMR
17	Terpinen-4-ol	1 161	1597	0.2	0.1	0.2	0.3	0.4	0.3	0.4	0.3	-	0.2	0.3	0.2	0.2	0.2	0.4	0.2	0.2	0.2	0.1	0.1	0.3	0.1	0.4	-	RI, MS, ^{13}C-NMR
18	α-Terpineol	1 171	1693	tr	tr	-	tr	0.3	0.1	0.1	0.1	-	tr	tr	tr	-	tr	0.1	-	-	tr	-	-	tr	-	tr	-	RI, MS
19	Citronellol	1 208	1761	0.4	0.1	0.1	0.1	0.1	tr	-	-	-	-	-	-	-	-	-	-	-	-	tr	tr	tr	tr	0.1	tr	RI, MS
20	Neral	1 212	1679	tr	-	-	tr	0.2	0.1	0.1	0.1	tr	tr	tr	0.1	0.1	0.1	0.2	tr	0.1	0.1	-	-	-	-	tr	-	RI, MS
21	Geraniol	1 233	1843	1.3	0.2	0.3	0.2	0.1	tr	tr	tr	-	-	-	-	-	-	tr	tr	tr	tr	0.2	0.1	0.2	0.1	0.2	0.1	RI, MS, ^{13}C-NMR
22	Lynalyl acetate	1 238	1553	-	-	-	-	0.6	0.3	0.2	0.1	-	-	-	0.1	-	0.1	0.2	-	-	0.1	-	-	-	-	-	-	RI, MS, ^{13}C-NMR
23	Geranial	1 244	1740	tr	tr	tr	tr	0.4	0.4	0.3	0.3	0.1	-	-	0.1	-	tr	0.1	-	-	0.1	0.1	0.1	-	0.1	-	0.1	RI, MS, ^{13}C-NMR
24	Thymol	1 267	2178	2.5	1.7	1.1	1.2	tr	tr	tr	tr	0.1	0.2	0.1	tr	0.1	-	tr	0.1	0.1	tr	tr	tr	0.1	tr	0.1	tr	RI, MS, ^{13}C-NMR
25	Carvacrol	1 277	2219	0.1	-	-	-	tr	-	-	-	-	tr	tr	-	-	tr	-	-	0.7	-	-	-	-	-	-	-	RI, MS, ^{13}C-NMR
26	Eugenol	1 328	2170	tr	-	-	-	-	-	-	-	-	-	-	-	-	-	-	-	0.4	tr	-	-	tr	tr	-	-	RI, MS, ^{13}C-NMR
27	Bicycloelemene	1 331	1485	tr	-	0.2	0.3	-	-	-	-	-	0.1	0.2	0.2	0.2	0.2	0.1	0.2	tr	0.2	tr	tr	0.2	0.1	0.2	-	RI, MS, ^{13}C-NMR
28	δ-Elemene	1 334	1464	0.5	-	2.8	4.0	0.1	0.2	0.4	tr	0.3	2.7	3.2	4.3	3.7	3.9	3.1	4.3	3.3	3.9	0.5	0.7	3.9	1.0	4.0	tr	RI, MS, ^{13}C-NMR
29	α-Cubebene	1 347	1452	-	-	tr	tr	0.1	0.1	0.1	0.1	0.1	0.1	0.1	0.1	0.1	0.1	0.1	0.1	tr	tr	0.1	0.1	0.1	0.1	0.1	0.1	RI, MS, ^{13}C-NMR
30	α-Ylangene	1 368	1475	tr	-	-	0.1	tr	tr	tr	tr	tr	0.1	0.1	0.1	0.1	0.1	0.1	0.1	0.2	0.1	tr	tr	tr	tr	tr	tr	RI, MS
31	α-Copaene	1 374	1485	0.1	-	0.3	0.1	1.1	1.1	1.0	1.2	1.2	0.3	0.4	0.3	0.4	0.2	0.5	0.4	0.7	0.3	0.7	0.7	0.1	0.7	0.1	0.9	RI, MS, ^{13}C-NMR
32	β-Cubebene	1 384	1 539	tr	0.7	-	0.1	-	-	-	-	-	tr	-	-	-	-	-	-	0.3	-	-	-	-	-	-	-	RI, MS, ^{13}C-NMR
33	β-Elemene	1 385	1583	0.6	tr	2.1	3.1	1.8	2.0	2.1	1.9	2.0	3.8	3.8	3.4	3.6	3.1	4.0	3.7	5.9	3.5	1.7	1.8	2.6	1.8	2.8	1.6	RI, MS, ^{13}C-NMR
34	α-Funebrene	1 386	1518	0.5	0.6	-	-	-	-	-	-	-	-	-	-	-	-	-	-	-	-	-	-	-	tr	-	-	RI, MS, ^{13}C-NMR
35	α-Gurjunene	1 398	1529	tr	-	-	0.1	tr	tr	tr	tr	tr	-	tr	-	tr	-	tr	tr	-	-	tr	tr	0.1	tr	0.1	tr	RI, MS, ^{13}C-NMR
36	Sesquithujene	1 400	1549	0.2	0.2	tr	-	-	-	-	-	-	-	tr	tr	-	tr	-	tr	-	tr	-	-	-	-	tr	-	RI, MS, ^{13}C-NMR
37	Cyperene	1 404	1524	-	-	0.3	-	-	tr	tr	-	tr	0.2	0.2	0.3	0.2	0.3	0.2	0.3	0.1	0.2	tr	tr	0.3	0.1	0.3	-	RI, MS, ^{13}C-NMR
38	*cis*-α-Bergamotene	1 409	1561	1.6	2.3	-	tr	tr	tr	tr	tr	tr	0.1	0.1	tr	-	tr	tr	tr	-	tr	-	-	tr	-	tr	-	RI, MS, ^{13}C-NMR
39	(*E*)-β-Caryophyllene*	1 416	1589	2.5	1.7	10.7	3.4	10.1	11.1	11.6	10.7	11.5	9.9	9.8	7.8	7.8	6.8	7.0	7.9	7.5	8.3	5.7	6.3	6.4	6.3	10.6	5.0	RI, MS, ^{13}C-NMR

40	α-Santalene*	1 416	1565	4.7	8.5	0.1	0.1	0.2	0.2	0.2	0.2	0.2	tr	tr	tr	tr	tr	tr	0.1	-	tr	0.1	-	tr	0.1	tr	0.1	RI, MS, ^{13}C-NMR
41	γ-Jasmolactone	1 419	2204	tr	-	0.1	0.2	-	-	-	-	-	0.1	0.1	0.1	0.1	0.1	0.1	0.1	0.1	0.1	-	-	0.1	tr	0.1	-	RI, MS
42	β-Copaene*	1 426	1 574	-	0.3	-	-	-	-	-	-	-	-	-	-	-	-	0.1	tr	0.6	-	-	-	-	-	-	-	RI, MS, ^{13}C-NMR
43	γ-Elemene*#	1 426	1 630	1.2	-	4.3	7.5	tr	tr	0.6	tr	0.5	7.9	7.4	7.1	7.2	6.9	6.1	7.3	6.2	7.1	0.5	0.9	1.4	1.1	4.3	tr	RI, MS, ^{13}C-NMR
44	*trans*-α-Bergamotene	1 431	1 578	8.9	11.7	0.1	tr	tr	tr	tr	tr	tr	-	-	-	-	-	-	-	-	-	-	-	-	-	tr	-	RI, MS, ^{13}C-NMR
45	Sesquisabinene A	1 434	1 636	0.5	1.3	tr	0.1	tr	tr	-	-	-	-	-	-	0.1	-	0.1	-	-	-	-	-	-	-	-	-	RI, MS, ^{13}C-NMR
46	β-Sesquifenchene	1 439	1 611	0.4	0.5	tr	tr	tr	tr	tr	tr	tr	-	0.1	-	-	-	-	-	-	-	tr	tr	-	tr	-	tr	RI, MS, ^{13}C-NMR
47	*epi*-β-Santalene	1 441	1 626	0.4	0.5	tr	0.1	tr	tr	tr	tr	tr	0.1	0.1	0.1	0.1	0.1	0.1	0.1	tr	0.1	tr	tr	tr	tr	tr	tr	RI, MS, ^{13}C-NMR
48	(*E*)-β-Farnesene	1 446	1 660	1.4	2.1	0.1	0.1	0.2	0.1	0.1	0.2	0.1	tr	tr	0.1	0.1	0.1	0.2	0.1	0.2	0.1	0.1	0.1	0.1	0.1	0.1	0.1	RI, MS, ^{13}C-NMR
49	α-Humulene	1 448	1 662	0.6	0.8	1.8	0.6	1.9	2.1	2.2	2.1	2.1	1.7	1.7	1.3	1.4	1.2	1.4	1.3	1.6	1.4	1.3	1.4	1.2	1.4	1.6	1.3	RI, MS, ^{13}C-NMR
50	β-Santalene	1 453	1 643	0.2	0.2	-	tr	-	0.2	-	-	-	-	-	-	-	-	-	-	-	-	tr	tr	-	tr	-	tr	RI, MS, *^{13}C-NMR*
51	(5αH,10βMe)-6,12-Oxido-elema-1,3,6,11(12)-tetraene#	1 455	1 837	-	-	10.3	tr	0.1	-	0.8	tr	0.5	6.4	5.7	6.5	6.4	10.3	5.0	6.3	3.2	5.2	1.0	1.5	10.6	2.2	9.8	0.1	RI, MS, ^{13}C-NMR
52	*allo*-Aromadendrene	1 457	1 639	tr	tr	tr	-	-	-	-	-	-	-	tr	-	tr	-	-	-	0.6	-	-	-	-	-	-	-	RI, MS, ^{13}C-NMR
53	Ishwarane	1 460	1 644	0.1	0.2	tr	-	tr	0.1	-	-	-	-	-	-	0.1	-	tr	0.1	0.5	tr	-	-	-	-	-	-	RI, MS, ^{13}C-NMR
54	β-Acoradiene	1 461	1 669	0.1	0.2	tr	-	-	-	-	-	-	-	-	-	-	-	-	-	-	-	-	-	-	-	-	-	RI, MS, *^{13}C-NMR*
55	6,12-Oxido-germacra-1(10),4,6,11(12)-tetraene#	1 463	1 845	-	-	1.2	-	-	-	tr	-	tr	0.6	0.5	0.5	0.5	1.0	1.4	0.5	0.1	0.4	0.1	0.2	1.1	0.2	0.9	-	RI, MS, ^{13}C-NMR
56	α-Curcumene	1 469	1 766	0.8	1.1	0.2	tr	0.1	0.5	0.4	0.5	tr	tr	0.3	0.2	0.3	0.2	0.3	0.3	0.4	0.2	tr	tr	0.2	tr	0.2	tr	RI, MS, ^{13}C-NMR
57	γ-Muurolene	1 471	1 683	0.7	0.8	1.4	0.2	0.6	-	-	-	0.4	2.7	2.6	2.2	2.3	2.5	2.7	2.3	3.9	2.2	0.3	0.3	1.7	0.3	1.7	0.3	RI, MS, ^{13}C-NMR
58	Germacrene D	1 474	1 700	2.6	0.2	11.1	17.8	27.6	27.1	22.6	29.2	28.8	4.9	5.6	7.8	8.1	7.5	5.1	7.6	0.9	6.8	20.5	21.7	4.0	20.6	3.8	23.6	RI, MS, ^{13}C-NMR
59	*trans*-β-Bergamotene	1 478	1 676	8.6	10.9	0.3	-	tr	0.1	0.1	tr	0.1	0.5	0.5	0.5	0.5	0.5	0.8	0.7	1.1	0.5	0.1	0.1	0.4	0.1	0.4	tr	RI, MS, ^{13}C-NMR
60	β-Selinene	1 484	1 710	0.1	0.1	tr	0.1	0.1	0.2	0.2	0.2	0.2	0.1	0.1	tr	0.1	0.1	0.1	0.1	0.1	0.1	tr	0.1	0.1	0.1	tr	0.1	RI, MS, ^{13}C-NMR
61	α-Zingiberene	1 485	1 712	0.4	0.4	tr	-	0.1	-	-	-	-	-	-	-	-	-	-	-	-	-	-	-	-	-	-	-	RI, MS, ^{13}C-NMR
62	α-Selinene	1 490	1 723	-	0.1	-	-	-	-	-	-	-	-	-	0.1	-	-	-	tr	0.7	-	-	-	-	-	tr	-	RI, MS, ^{13}C-NMR
63	Bicyclogermacrene*	1 491	1 721	0.2	0.1	1.1	0.2	1.6	1.6	1.4	1.6	1.7	0.4	0.4	0.5	0.6	0.1	0.4	0.5	0.2	0.5	1.6	1.6	0.3	1.5	0.3	1.8	RI, MS, ^{13}C-NMR
64	α-Muurolene*	1 491	1 720	-	tr	-	-	-	-	-	-	tr	-	-	-	-	-	tr	tr	0.3	-	-	-	-	-	-	-	RI, MS, ^{13}C-NMR
65	(*Z*)-α-Bisabolene	1 492	1 724	0.2	0.1	-	0.3	0.4	0.7	0.4	0.4	0.5	tr	0.1	0.1	0.1	0.1	tr	0.1	-	-	-	-	-	-	-	-	RI, MS, ^{13}C-NMR
66	γ-Cadinene	1 493	1 753	tr	-	-	0.3	0.5	0.4	0.4	0.5	0.4	0.2	0.2	0.2	0.1	0.1	0.2	0.2	0.2	0.2	0.2	0.3	0.2	0.3	0.5	0.2	RI, MS, ^{13}C-NMR
67	β-Bisabolene	1 500	1 719	6.8	8.7	0.1	tr	0.4	0.3	0.3	0.3	0.2	0.1	0.1	0.1	0.1	0.1	0.2	0.1	0.2	0.1	0.2	0.2	0.1	0.2	0.2	0.2	RI, MS, ^{13}C-NMR
68	(*E*,*E*)-α-Farnesene	1 501	1 748	0.7	0.7	0.3	0.2	0.3	0.3	-	-	0.1	0.4	0.4	0.3	0.3	0.3	0.2	0.4	0.1	0.3	tr	0.1	0.3	0.1	0.2	-	RI, MS, ^{13}C-NMR
69	β-Curcumene	1 502	1 736	0.3	0.4	-	tr	-	-	-	-	-	-	-	-	-	-	-	-	0.3	-	-	-	-	-	-	-	RI, MS, *^{13}C-NMR*
70	(*Z*)-γ-Curcumene	1 506	1 732	0.6	0.8	-	tr	0.2	0.1	0.3	0.1	0.3	-	-	0.2	0.4	0.2	0.3	0.2	-	0.2	-	-	0.2	-	-	-	RI, MS, ^{13}C-NMR
71	β-Sesquiphellandrene	1 512	1 766	0.4	0.4	0.2	0.3	0.1	-	-	-	-	-	-	-	-	-	-	-	-	-	-	-	-	-	-	-	RI, MS, *^{13}C-NMR*
72	δ-Cadinene	1 514	1 753	tr	-	0.2	0.2	2.4	0.2	2.1	2.4	2.3	0.1	0.2	0.2	tr	tr	tr	0.2	0.1	0.2	2.4	2.2	0.1	1.0	0.1	2.5	RI, MS, ^{13}C-NMR
73	*cis*-Lanceol	1 517	2 087	0.3	0.3	tr	tr	0.6	-	0.5	0.5	0.6	tr	tr	tr	tr	tr	1.0	tr	1.7	tr	0.7	0.9	tr	tr	tr	0.9	RI, MS, ^{13}C-NMR
74	Cadina-1,4-diene	1 519	1 762	-	-	0.2	tr	0.8	0.8	0.8	0.9	0.7	tr	tr	tr	tr	tr	-	tr	-	tr	tr	tr	tr	1.4	tr	-	RI, MS, ^{13}C-NMR
75	(*Z*)-γ-Bisabolene	1 521	1 721	0.7	0.9	0.4	tr	0.1	-	0.1	tr	-	0.7	0.7	0.5	0.7	0.6	0.3	0.6	0.4	0.6	1.5	1.4	0.4	tr	0.4	1.4	RI, MS, ^{13}C-NMR
76	*trans*-Sesquisabinene hydrate	1 530	1 984	1.8	2.0	0.1	tr	tr	tr	tr	0.1	tr	0.4	0.5	0.3	0.3	0.3	0.4	0.4	0.6	0.3	0.1	tr	0.2	tr	0.2	tr	RI, MS, ^{13}C-NMR
77	(*E*)-α-Bisabolene	1 531	1 761	0.8	0.6	0.3	0.6	tr	0.1	-	-	-	tr	tr	tr	tr	tr	tr	tr	-	tr	-	-	-	-	-	-	RI, MS, ^{13}C-NMR
78	(10βH)-1β,8β-Oxido-cadina-4-ene	1 534	1 853	8.7	9.3	1.6	-	6.8	6.4	5.7	6.4	5.8	0.2	0.2	0.2	0.9	0.4	0.4	0.3	0.5	0.2	6.7	6.6	0.2	6.1	0.4	7.3	RI, MS, ^{13}C-NMR
79	β-Elemol	1 536	2 077	0.4	0.4	0.5	tr	-	-	-	-	tr	0.5	0.5	0.4	0.7	0.7	0.5	0.7	0.7	0.7	0.1	0.1	0.5	0.2	0.8	tr	RI, MS, ^{13}C-NMR
80	(*E*)-Nerolidol	1 547	2 034	1.9	1.1	0.2	0.1	0.5	-	-	-	0.8	0.1	tr	0.1	tr	0.1	0.3	0.1	tr	0.1	1.1	1.1	tr	1.0	tr	0.5	RI, MS, ^{13}C-NMR
81	Germacrene B#	1 549	1 818	2.8	-	14.2	24.8	0.5	1.7	2.4	0.5	2.0	24.5	22.3	21.1	20.7	20.8	17.4	21.7	14.0	22.0	2.4	3.1	17.2	4.0	12.6	0.4	RI, MS, ^{13}C-NMR

82	Palustrol	1 559	1 924	-	-	tr	-	-	-	-	0.1	tr	tr	-	-	-	-	-	0.1	0.6	tr	-	tr	-	-	-	-	RI, MS, ^{13}C-NMR
83	Santalenone	1 560	1 980	10.7	13.0	0.1	tr	-	-	-	-	-	tr	0.1	tr	tr	tr	tr	-	-	-	-	-	-	-	-	-	RI, MS, ^{13}C-NMR
84	*cis*-Sesquisabinene hydrate	1 562	2 079	0.8	0.8	tr	0.1	0.2	0.2	0.3	0.2	0.2	tr	tr	tr	tr	tr	tr	0.1	0.3	tr	0.2	0.2	tr	0.2	tr	0.3	RI, MS, ^{13}C-NMR
85	Germacra-1(10),5-dien-4β-ol	1 564	2 047	0.9	0.1	tr	-	-	-	-	tr	-	-	-	-	-	tr	-	-	0.3	0.1	-	-	-	-	tr	tr	RI, MS, ^{13}C-NMR
86	Caryophyllene oxide	1 567	1 973	0.2	0.3	0.3	0.2	0.1	0.6	0.9	0.7	0.4	1.4	1.6	1.2	2.0	0.8	5.1	1.6	11.5	1.3	0.2	0.1	0.9	0.3	0.7	0.1	RI, MS, ^{13}C-NMR
87	Guaiol	1 581	2 119	1.7	2.3	0.8	0.2	tr	tr	-	-	-	tr	tr	tr	0.2	tr	0.3	tr	tr	tr	tr	tr	tr	tr	tr	tr	RI, MS, ^{13}C-NMR
88	Germacrene-D-8-one	1 584	2 066	0.3	0.5	0.2	0.1	4.1	3.9	3.2	3.8	4.2	-	tr	-	0.2	0.1	0.1	tr	0.3	tr	8.7	7.3	tr	7.6	tr	8.7	RI, MS, ^{13}C-NMR
89	Ledol	1 591	2 024	0.1	tr	0.1	-	tr	-	-	-	-	-	tr	tr	-	-	-	-	0.5	tr	-	-	-	-	-	-	RI, MS, ^{13}C-NMR
90	Humulene oxide II	1 597	2 042	0.3	0.1	0.2	0.6	0.3	tr	0.3	0.1	0.1	tr	0.1	tr	tr	tr	0.7	tr	1.5	0.2	0.2	0.1	0.1	0.2	0.1	0.4	RI, MS, ^{13}C-NMR
91	*epi*-Cubenol	1 605	2 046	0.2	0.2	0.2	0.5	tr	tr	tr	tr	tr	0.4	0.2	0.2	0.3	0.3	0.2	0.2	0.2	0.3	-	-	0.2	tr	0.1	-	RI, MS, ^{13}C-NMR
92	Alismol	1 609	2 245	0.2	-	0.2	0.9	0.1	tr	0.1	tr	0.1	1.4	1.1	0.7	1.0	0.6	1.5	1.0	3.4	1.2	tr	0.1	0.3	0.1	0.4	0.1	RI, MS, ^{13}C-NMR
93	Eremoligenol	1 614	2 196	0.3	0.3	0.1	-	-	-	-	-	-	-	-	-	-	-	-	-	0.6	tr	0.1	-	-	-	-	-	RI, MS, ^{13}C-NMR
94	Cubenol	1 619	2 064	0.1	tr	0.6	tr	-	-	-	-	-	-	-	-	-	-	-	-	0.4	-	-	-	-	-	-	-	RI, MS, ^{13}C-NMR
95	γ-Eudesmol	1 620	2 172	0.1	tr	1.0	0.5	0.6	0.8	0.6	0.6	0.5	0.9	0.6	0.6	0.7	0.9	0.6	0.7	0.3	0.7	1.2	1.1	0.4	1.2	0.3	1.2	RI, MS, ^{13}C-NMR
96	δ-Cadinol	1 626	2 174	tr	tr	0.1	tr	-	0.1	tr	-	tr	0.1	0.1	0.1	tr	tr	tr	0.1	0.2	tr	0.1	0.1	-	0.1	-	0.1	RI, MS
97	Muurola-4,10(14)-dien-8β-ol	1 629	2 186	1.0	0.9	0.9	tr	2.8	2.6	2.3	2.7	2.2	0.1	0.1	0.1	0.3	0.1	0.1	0.1	0.2	0.1	2.9	2.7	tr	2.5	tr	3.2	RI, MS, ^{13}C-NMR
98	β-Eudesmol	1 635	2 225	-	tr	-	-	-	-	-	-	-	-	-	-	-	0.2	tr	tr	0.1	-	-	-	-	-	-	-	RI, MS, ^{13}C-NMR
99	α-Cadinol	1 637	2 231	0.4	0.2	0.9	0.1	tr	0.3	0.5	0.6	0.5	0.7	0.7	0.6	0.8	0.7	0.7	0.8	0.8	0.6	0.6	0.6	0.5	0.6	0.7	0.6	RI, MS, ^{13}C-NMR
100	α-Eudesmol	1 639	2 216	-	-	-	-	-	-	-	-	-	-	-	-	-	0.1	tr	tr	0.1	-	-	-	-	-	-	-	RI, MS, ^{13}C-NMR
101	Tumerone-ar	1 642	2 252	-	-	tr	0.1	tr	-	-	-	-	-	-	-	-	-	-	tr	0.3	tr	-	-	-	-	-	0.1	RI, MS, ^{13}C-NMR
102	β-Bisabolol	1 653	2 144	1.3	1.1	-	tr	0.1	0.1	tr	tr	tr	-	-	-	0.1	0.1	0.2	0.2	0.3	-	0.2	0.3	0.1	0.2	0.2	0.2	RI, MS, ^{13}C-NMR
103	(7βH)-Germacrene D-8α-ol*	1 657	2 355	tr	tr	1.4	1.5	2.5	3.4	2.0	2.2	2.0	0.2	tr	0.1	-	-	-	-	0.3	0.2	7.4	6.0	tr	6.1	-	7.8	RI, MS, ^{13}C-NMR
104	(7βH)-Germacrene D-8β-ol*	1 657	2 355	tr	tr	0.5	0.5	0.8	1.2	0.7	0.7	1.1	tr	tr	tr	-	-	-	-	tr	tr	2.5	2.1	tr	2.3	-	2.6	RI, MS, ^{13}C-NMR
105	α-Bisabolol	1 664	2 234	0.7	0.7	tr	tr	0.9	0.9	0.7	0.7	0.7	tr	0.1	tr	tr	tr	0.2	tr	tr	tr	1.5	1.1	tr	1.1	tr	1.4	RI, MS, ^{13}C-NMR
106	*epi*-α-Bisabolol	1 667	2 208	0.6	0.7	tr	tr	0.1	0.1	0.1	-	0.1	0.1	tr	tr	tr	tr	tr	tr	0.1	tr	tr	0.1	tr	tr	-	0.1	RI, MS, ^{13}C-NMR
107	Germacrone	1 671	2 219	0.3	-	tr	3.5	-	-	-	0.1	-	-	-	-	-	tr	-	tr	-	-	-	-	-	tr	-	-	RI, MS, ^{13}C-NMR
108	Cadina-1(10),4-dien-8α-ol	1 672	2 306	0.1	-	-	tr	-	-	-	tr	-	0.1	0.1	-	-	-	0.1	-	0.2	-	tr	-	-	-	-	-	RI, MS, ^{13}C-NMR
109	Cadina-1(10),4-dien-8β-ol	1 676	2 276	0.2	tr	2.1	0.2	5.3	5.0	4.2	4.6	4.1	0.2	0.2	0.2	0.7	0.3	0.3	0.2	0.3	0.2	7.2	6.4	0.1	6.2	0.2	7.6	RI, MS, ^{13}C-NMR
110	Cadina-4,10(14)-dien-8β-ol	1 678	2 280	0.1	0.1	0.5	0.1	0.4	tr	0.3	0.1	0.1	0.4	0.1	0.3	0.4	0.4	0.3	0.1	0.1	0.1	0.8	0.4	0.2	0.8	0.4	0.8	RI, MS, ^{13}C-NMR
111	(1βH,5βH)-6,12-Oxido-guaia-6,10(14),11(12)-trien-4α-ol	1 754	2 519	tr	tr	0.5	-	-	0.1	0.2	-	tr	2.2	1.4	1.2	1.8	1.7	2.3	1.3	5.1	2.0	0.1	0.1	0.3	0.1	0.6	-	RI, MS, ^{13}C-NMR
112	(6αH)-Germacra-1(10),4,7(11)-trien-6,12-γ-lactone	1 856	2 829	-	-	tr	-	-	-	-	-	-	0.2	0.1	0.1	0.2	0.8	0.2	0.1	0.6	0.2	-	-	tr	-	0.1	-	RI, MS, ^{13}C-NMR
113	(*E*)-Phytol	2 097	2 609	-	-	-	-	tr	-	tr	-	tr	-	0.1	tr	0.1	-	tr	0.1	0.1	tr	-	-	-	-	-	-	RI, MS
	Hydrocarbon monoterpenes			5.4	3.2	16.6	21.4	14.0	15.8	19.5	15.9	15.7	15.0	19.2	21.9	16.7	19.9	18.3	18.3	6.2	20.6	11.4	12.4	38.8	13.6	35.2	10.0	
	Oxygenated monoterpenes			4.6	2.2	1.8	1.9	3.7	1.8	1.5	1.3	0.3	0.5	0.5	0.7	0.5	0.6	1.5	0.4	1.3	0.7	0.5	0.4	0.8	0.4	1.0	0.2	
	Hydrocarbon sesquiterpenes			51.2	58.1	52.8	64.7	51.3	51.3	49.8	53.0	55.7	61.6	60.7	59.1	59.4	56.1	51.1	61.0	50.6	59.1	39.9	43.1	41.6	42.4	44.6	39.6	
	Oxygenated Sesquiterpenes			33.7	34.4	24.6	9.2	26.3	25.7	23.4	24.2	24.0	16.6	14.0	13.4	17.5	19.9	21.9	14.9	35.4	14.1	43.7	39.2	15.7	39.3	15.9	44.1	
	Other compounds			0.0	0.0	0.1	0.2	0.0	0.0	0.0	0.0	0.0	0.1	0.2	0.1	0.2	0.1	0.1	0.2	0.6	0.1	0.0	0.0	0.1	0.0	0.1	0.0	
	**Total**			**94.9**	**97.9**	**95.9**	**97.4**	**95.3**	**94.6**	**94.2**	**94.4**	**95.7**	**93.8**	**94.6**	**95.2**	**94.3**	**96.6**	**92.9**	**94.8**	**94.1**	**94.6**	**95.5**	**95.1**	**97.0**	**95.7**	**96.8**	**93.9**	

Order of elution and percentages are given on an apolar column (BP-1), except components with an asterisk (*), where percentages are taken on a polar column (BP-20). (#) Thermolabile compound, percentage evaluated by a combination of GC-FID and ^{13}C-NMR data. RIa, RIp: retention indices measured on apolar and polar capillary column, respectively. tr: traces level (<0.05%). ^{13}C-NMR: compounds identified by NMR in the essential oil samples and obvious in at least one fraction of chromatography; *^{13}C-NMR (italic)*: compounds identified by NMR in fractions of chromatography.

Table S1 (Continuation). Chemical composition of the 47 leaf essential oil samples from *Isolona dewevrei* (Samples 25 to 47).

	Compounds	RIa	RIp	25	26	27	28	29	30	31	32	33	34	35	36	37	38	39	40	41	42	43	44	45	46	47	Identification
1	α-Thujene	923	1016	0.4	0.6	1.3	0.6	0.7	0.1	0.2	0.3	0.3	0.5	0.1	0.6	0.2	0.5	0.1	0.1	0.4	0.1	0.4	0.1	0.6	0.4	0.2	RI, MS, ^{13}C-NMR
2	α-Pinene	931	1013	0.2	0.5	1.5	0.3	0.4	0.1	0.3	0.3	0.3	0.3	tr	0.2	0.1	0.2	tr	tr	0.1	-	0.1	tr	0.2	0.1	0.1	RI, MS, ^{13}C-NMR
3	Sabinene	965	1120	1.6	2.0	5.0	2.4	3.0	0.4	0.8	1.4	0.7	2.0	0.5	2.0	0.7	1.9	0.3	0.1	2.0	0.1	2.0	0.2	2.4	1.8	1.0	RI, MS, ^{13}C-NMR
4	β-Pinene	970	1109	0.2	0.6	1.8	0.3	0.4	0.2	0.5	0.4	0.4	0.2	tr	0.1	tr	0.1	-	-	tr	-	tr	-	0.1	tr	-	RI, MS, ^{13}C-NMR
5	Myrcene	981	1158	0.5	1.0	1.7	0.6	0.8	0.3	0.1	0.2	tr	0.7	0.2	0.6	0.4	0.5	0.3	0.2	0.5	0.2	0.4	0.9	0.5	0.5	0.3	RI, MS, ^{13}C-NMR
6	α-Phellandrene	997	1162	tr	0.1	0.1	tr	0.1	tr	-	0.1	-	tr	tr	0.1	tr	tr	tr	tr	tr	tr	tr	0.1	tr	tr	-	RI, MS
7	δ-3-Carene	1 005	1146	tr	0.1	0.1	tr	tr	tr	tr	tr	tr	tr	tr	tr	-	tr	tr	tr	tr	tr	0.1	0.1	0.1	tr	-	RI, MS
8	α-Terpinene	1 009	1178	0.1	0.3	0.4	0.2	0.2	0.1	-	-	-	0.2	0.4	0.2	0.1	0.2	tr	tr	0.1	tr	tr	0.2	tr	0.1	tr	RI, MS, ^{13}C-NMR
9	*p*-Cymene	1 012	1268	0.1	0.3	0.4	0.1	0.2	0.1	1.5	0.9	1.0	0.2	tr	0.1	0.1	tr	tr	tr	tr	tr	-	0.1	-	tr	-	RI, MS, ^{13}C-NMR
10	Limonene	1 021	1199	1.2	3.2	13.0	1.8	2.6	1.2	1.8	0.9	0.8	1.7	0.2	0.4	0.1	0.4	tr	tr	0.1	tr	0.1	0.2	0.1	0.1	tr	RI, MS, ^{13}C-NMR
11	(*Z*)-β-Ocimene	1 025	1230	8.8	12.5	1.4	9.0	9.2	4.3	0.4	1.1	0.2	13.8	7.8	13.5	12.8	14.0	10.6	10.3	13.7	9.4	13.5	36.6	11.5	13.6	10.2	RI, MS, ^{13}C-NMR
12	(*E*)-β-Ocimene	1 036	1247	6.4	9.7	2.5	3.9	5.1	4.1	0.3	0.8	0.2	5.2	4.4	4.9	5.9	5.5	7.6	8.3	9.1	7.9	5.9	30.5	5.3	6.5	3.4	RI, MS, ^{13}C-NMR
13	γ-Terpinene	1 048	1242	0.4	0.6	1.0	0.4	0.5	0.2	-	-	-	0.3	0.1	0.5	0.2	0.3	0.1	0.1	0.2	0.1	0.2	0.5	0.2	0.2	0.1	RI, MS, ^{13}C-NMR
14	Terpinolene	1 078	1279	0.1	0.2	0.2	0.1	0.1	tr	tr	0.1	-	0.1	tr	0.1	0.1	0.1	tr	tr	tr	tr	0.1	0.1	tr	0.1	-	RI, MS
15	Linalool	1 083	1543	0.1	0.1	0.1	0.1	0.1	0.1	0.3	0.2	0.2	0.1	0.1	0.1	0.1	0.1	0.1	0.1	0.1	0.1	0.1	0.1	0.1	0.1	tr	RI, MS, ^{13}C-NMR
16	*allo*-Ocimene	1 117	1370	0.3	0.3	tr	0.2	0.2	0.1	0.1	-	-	0.4	0.3	0.4	0.4	0.5	0.4	0.4	0.5	0.3	0.5	1.3	0.4	0.5	0.4	RI, MS, ^{13}C-NMR
17	Terpinen-4-ol	1 161	1597	0.1	0.2	0.3	0.3	0.3	tr	0.1	0.1	0.1	0.2	0.1	0.3	0.2	0.3	tr	tr	0.2	-	0.2	tr	0.2	0.1	0.1	RI, MS, ^{13}C-NMR
18	α-Terpineol	1 171	1693	tr	tr	tr	tr	tr	-	-	-	-	-	-	-	-	-	-	-	tr	-	tr	-	tr	-	-	RI, MS
19	Citronellol	1 208	1761	tr	tr	tr	tr	tr	-	-	-	-	-	-	-	-	-	-	-	-	-	-	-	-	-	-	RI, MS
20	Neral	1 212	1679	-	tr	0.1	-	tr	-	tr	-	-	-	tr	-	tr	-	tr	-	0.3	-	0.2	tr	-	-	0.1	RI, MS
21	Geraniol	1 233	1843	0.1	0.1	0.1	0.1	0.1	tr	tr	-	tr	-	0.1	-	0.1	-	0.1	-	0.1	-	tr	tr	-	-	tr	RI, MS, ^{13}C-NMR
22	Lynalyl acetate	1 238	1553	-	-	-	-	-	-	-	-	-	-	-	-	-	-	-	-	-	-	-	tr	-	-	-	RI, MS, ^{13}C-NMR
23	Geranial	1 244	1740	0.1	0.1	-	-	-	0.1	0.3	0.5	0.5	tr	tr	-	-	-	-	-	0.4	-	0.3	-	-	-	0.1	RI, MS, ^{13}C-NMR
24	Thymol	1 267	2178	tr	tr	tr	tr	0.1	tr	0.3	-	-	0.1	1.1	tr	0.7	tr	0.8	0.1	0.1	0.1	tr	0.2	0.1	0.1	-	RI, MS, ^{13}C-NMR
25	Carvacrol	1 277	2219	-	-	-	-	-	-	0.5	0.2	0.3	tr	-	-	-	-	-	-	tr	-	-	-	-	-	1.0	RI, MS, ^{13}C-NMR
26	Eugenol	1 328	2170	-	-	-	tr	-	-	0.1	0.3	0.1	-	-	-	-	-	-	-	-	-	0.7	-	-	-	0.4	RI, MS, ^{13}C-NMR
27	Bicycloelemene	1 331	1485	0.1	0.1	0.2	0.3	0.3	tr	-	-	-	0.2	0.1	-	0.1	-	0.1	-	0.1	tr	0.1	-	0.1	0.1	0.1	RI, MS, ^{13}C-NMR
28	δ-Elemene	1 334	1464	0.9	1.5	4.1	4.3	4.5	0.6	1.2	0.7	0.5	4.2	2.6	0.3	2.3	0.4	0.9	0.3	1.1	0.5	1.5	0.3	1.3	1.6	1.9	RI, MS, ^{13}C-NMR
29	α-Cubebene	1 347	1452	0.1	0.1	tr	tr	0.1	0.1	0.3	0.1	0.2	tr	0.1	0.1	0.1	0.1	0.1	0.1	tr	0.1	0.1	0.2	-	tr	-	RI, MS, ^{13}C-NMR
30	α-Ylangene	1 368	1475	tr	tr	tr	tr	tr	tr	0.1	-	tr	0.1	tr	tr	tr	tr	tr	tr	tr	tr	0.1	0.1	-	tr	0.1	RI, MS
31	α-Copaene	1 374	1485	0.8	0.6	0.2	0.1	0.1	0.7	2.8	1.4	2.6	0.3	0.3	0.9	0.4	0.6	0.6	0.7	0.3	0.7	0.5	0.8	0.2	0.7	0.1	RI, MS, ^{13}C-NMR
32	β-Cubebene	1 384	1 539	-	-	-	-	-	-	0.2	0.5	0.4	0.1	0.1	0.3	0.2	0.3	0.2	0.2	0.1	0.2	0.3	0.3	0.2	0.5	0.1	RI, MS, ^{13}C-NMR
33	β-Elemene	1 385	1583	1.8	2.2	2.7	2.7	2.8	1.7	2.4	2.2	2.8	3.2	3.2	1.9	3.3	1.5	1.9	1.4	1.9	1.6	1.7	1.3	2.2	4.0	1.2	RI, MS, ^{13}C-NMR
34	α-Funebrene	1 386	1518	-	-	-	tr	-	-	-	-	-	-	-	-	-	-	-	-	-	-	-	tr	-	-	-	RI, MS, ^{13}C-NMR
35	α-Gurjunene	1 398	1529	tr	0.1	tr	-	tr	tr	tr	1.3	-	tr	-	-	-	-	-	-	1.0	-	0.4	-	1.1	-	-	RI, MS, ^{13}C-NMR
36	Sesquithujene	1 400	1549	-	-	tr	tr	tr	-	tr	-	-	-	-	tr	-	-	-	-	-	-	-	tr	-	-	-	RI, MS, ^{13}C-NMR
37	Cyperene	1 404	1524	0.1	0.1	0.4	0.3	0.3	tr	-	-	-	0.1	-	-	0.2	-	0.1	-	tr	0.2	-	-	-	-	tr	RI, MS, ^{13}C-NMR
38	*cis*-α-Bergamotene	1 409	1561	-	-	-	tr	tr	-	-	-	-	tr	tr	-	-	-	-	-	-	-	-	-	-	-	-	RI, MS, ^{13}C-NMR
39	(*E*)-β-Caryophyllene*	1 416	1589	5.8	5.3	11.3	10.7	12.1	5.7	3.6	3.1	3.8	7.9	8.6	5.6	8.3	5.2	4.1	2.6	8.1	3.1	7.5	2.2	6.9	10.4	12.4	RI, MS, ^{13}C-NMR

40	α-Santalene*	1 416	1565	0.1	0.1	tr	tr	tr	0.1	4.1	4.2	4.6	0.1	-	-	-	-	-	-	-	-	-	-	-	-	-	RI, MS, ^{13}C-NMR
41	γ-Jasmolactone	1 419	2204	-	tr	0.1	0.1	0.1	-	-	-	-	0.1	-	-	-	-	-	-	-	-	-	-	-	-	-	RI, MS
42	β-Copaene*	1 426	1 574	-	-	-	-	-	-	0.3	0.5	0.7	0.1	0.2	0.2	0.1	0.3	0.2	0.2	0.2	0.2	0.2	0.2	0.1	0.1	0.1	RI, MS, ^{13}C-NMR
43	γ-Elemene*#	1 426	1 630	0.6	1.6	4.0	5.0	5.0	0.6	1.2	1.3	0.5	6.3	3.8	0.4	2.1	0.3	0.5	0.1	0.4	0.5	0.4	tr	0.5	0.5	0.3	RI, MS, ^{13}C-NMR
44	*trans*-α-Bergamotene	1 431	1 578	-	-	tr	-	-	-	6.1	6.0	6.2	tr	-	tr	-	-	-	-	-	-	-	-	-	-	-	RI, MS, ^{13}C-NMR
45	Sesquisabinene A	1 434	1 636	-	-	-	-	tr	-	0.1	-	tr	0.1	-	-	-	-	-	-	-	-	-	-	-	-	tr	RI, MS, ^{13}C-NMR
46	β-Sesquifenchene	1 439	1 611	tr	tr	-	-	-	tr	-	-	-	-	-	-	-	-	-	-	-	-	-	-	-	-	-	RI, MS, ^{13}C-NMR
47	*epi*-β-Santalene	1 441	1 626	tr	tr	tr	0.1	0.1	tr	-	-	-	0.1	-	-	-	-	-	-	-	-	-	-	-	-	-	RI, MS, ^{13}C-NMR
48	(*E*)-β-Farnesene	1 446	1 660	0.1	0.1	0.1	0.1	0.1	0.1	-	-	-	0.1	0.1	-	-	-	-	0.1	-	0.1	-	tr	-	-	-	RI, MS, ^{13}C-NMR
49	α-Humulene	1 448	1 662	1.2	1.4	1.8	1.8	1.9	1.4	2.7	2.2	2.5	1.3	0.5	1.2	1.7	1.1	1.0	0.8	2.7	0.8	2.0	0.5	2.8	2.9	1.9	RI, MS, ^{13}C-NMR
50	β-Santalene	1 453	1 643	-	-	-	-	-	-	-	-	-	-	-	-	-	-	-	-	-	-	-	-	-	-	-	RI, MS, *^{13}C-NMR*
51	(5αH,10βMe)-6,12-Oxido-elema-1,3,6,11(12)-tetraene#	1 455	1 837	1.6	3.9	11.6	13.7	10.7	1.5	1.1	1.4	0.6	2.0	8.8	0.7	5.8	0.7	2.0	0.4	0.9	0.5	2.2	-	1.7	1.6	1.8	RI, MS, ^{13}C-NMR
52	*allo*-Aromadendrene	1 457	1 639	-	-	-	tr	-	-	0.4	0.6	0.5	-	-	-	-	-	-	tr	1.2	-	-	tr	1.3	-	-	RI, MS, ^{13}C-NMR
53	Ishwarane	1 460	1 644	-	tr	-	-	-	-	0.6	0.1	0.7	-	-	-	-	-	-	-	0.1	-	-	-	tr	0.8	-	RI, MS, ^{13}C-NMR
54	β-Acoradiene	1 461	1 669	-	-	-	-	-	-	-	-	-	-	0.1	-	-	-	-	-	-	-	-	tr	-	-	-	RI, MS, *^{13}C-NMR*
55	6,12-Oxido-germacra-1(10),4,6,11(12)-tetraene#	1 463	1 845	0.2	0.4	1.2	1.6	1.2	0.1	-	-	0.8	0.2	0.8	tr	0.5	tr	0.2	-	0.1	tr	0.2	-	0.1	0.2	0.2	RI, MS, ^{13}C-NMR
56	α-Curcumene	1 469	1 766	tr	0.3	tr	0.2	0.2	tr	1.2	0.5	0.9	0.2	0.1	-	-	-	-	0.1	-	0.1	-	tr	-	-	-	RI, MS, ^{13}C-NMR
57	γ-Muurolene	1 471	1 683	0.3	0.8	1.7	2.0	1.9	0.3	1.1	0.6	0.4	0.4	0.4	0.7	0.5	0.6	0.4	0.5	0.4	0.4	0.9	0.3	0.7	2.5	0.2	RI, MS, ^{13}C-NMR
58	Germacrene D	1 474	1 700	21.7	18.5	4.8	6.0	5.6	21.3	1.0	2.8	1.7	15.3	16.9	29.7	16.4	31.4	23.1	25.8	24.4	24.5	34.0	13.8	10.3	9.9	16.3	RI, MS, ^{13}C-NMR
59	*trans*-β-Bergamotene	1 478	1 676	tr	0.2	0.4	0.4	0.4	0.1	6.2	7.0	6.1	0.5	0.1	-	-	-	-	0.1	-	0.2	-	-	-	-	-	RI, MS, ^{13}C-NMR
60	β-Selinene	1 484	1 710	0.1	0.1	tr	0.1	0.1	0.1	0.2	0.1	0.2	0.1	0.4	tr	tr	tr	0.1	-	0.8	tr	0.7	tr	1.3	3.1	1.8	RI, MS, ^{13}C-NMR
61	α-Zingiberene	1 485	1 712	-	-	tr	-	-	-	-	-	-	0.5	0.1	-	-	-	-	0.1	-	0.1	-	0.1	-	-	-	RI, MS, ^{13}C-NMR
62	α-Selinene	1 490	1 723	-	-	-	-	-	tr	0.9	0.5	0.8	-	-	-	-	-	-	-	1.6	-	1.1	-	2.0	1.2	1.4	RI, MS, ^{13}C-NMR
63	Bicyclogermacrene*	1 491	1 721	1.5	1.1	tr	0.2	0.4	1.6	0.3	0.5	0.3	0.2	0.9	1.6	1.4	1.2	1.1	1.3	-	1.3	-	0.7	-	-	-	RI, MS, ^{13}C-NMR
64	α-Muurolene*	1 491	1 720	-	-	-	-	-	tr	0.5	0.6	0.5	-	0.4	0.4	-	0.4	0.3	0.3	0.4	0.3	0.4	0.2	0.4	0.1	0.1	RI, MS, ^{13}C-NMR
65	(*Z*)-α-Bisabolene	1 492	1 724	-	-	-	-	-	-	-	-	-	0.2	-	-	-	-	-	-	-	-	-	-	-	-	-	RI, MS, ^{13}C-NMR
66	γ-Cadinene	1 493	1 753	0.3	0.3	0.4	-	0.3	0.3	0.9	0.5	0.7	0.2	0.2	0.3	0.3	0.4	0.2	0.3	0.4	0.2	0.3	0.1	0.5	tr	0.1	RI, MS, ^{13}C-NMR
67	β-Bisabolene	1 500	1 719	0.2	0.2	0.1	0.1	0.1	0.2	3.4	3.6	2.6	0.1	-	-	-	-	-	0.1	-	0.5	-	-	-	-	-	RI, MS, ^{13}C-NMR
68	(*E*,*E*)-α-Farnesene	1 501	1 748	0.1	0.2	0.2	0.4	0.4	tr	0.1	0.1	-	0.1	-	-	-	-	-	-	-	-	-	-	-	-	-	RI, MS, ^{13}C-NMR
69	β-Curcumene	1 502	1 736	-	-	0.2	0.2	0.2	-	-	-	-	-	-	-	-	-	-	-	-	-	-	-	-	-	-	RI, MS, *^{13}C-NMR*
70	(*Z*)-γ-Curcumene	1 506	1 732	-	-	-	-	-	-	-	-	-	0.1	-	-	-	-	-	-	-	-	-	-	-	-	-	RI, MS, ^{13}C-NMR
71	β-Sesquiphellandrene	1 512	1 766	-	-	-	-	-	-	-	-	-	-	-	-	-	-	-	-	-	-	-	-	-	-	-	RI, MS, *^{13}C-NMR*
72	δ-Cadinene	1 514	1 753	2.0	1.4	0.1	0.2	0.1	2.3	2.0	2.9	2.6	0.6	0.8	1.4	1.4	2.2	1.9	2.3	1.6	2.1	1.1	0.8	1.7	0.3	0.3	RI, MS, ^{13}C-NMR
73	*cis*-Lanceol	1 517	2 087	0.7	0.5	tr	tr	tr	0.8	0.8	0.7	0.7	0.2	-	-	-	-	-	-	-	-	-	0.2	-	-	-	RI, MS, ^{13}C-NMR
74	Cadina-1,4-diene	1 519	1 762	-	0.8	tr	-	tr	-	tr	0.2	-	0.2	-	-	-	-	-	-	-	-	-	-	-	-	-	RI, MS, ^{13}C-NMR
75	(*Z*)-γ-Bisabolene	1 521	1 721	1.2	0.2	0.5	0.6	0.5	1.4	0.1	tr	-	tr	-	-	-	-	-	-	-	-	-	0.2	-	-	-	RI, MS, ^{13}C-NMR
76	*trans*-Sesquisabinene hydrate	1 530	1 984	0.1	tr	tr	tr	tr	tr	tr	tr	tr	0.2	-	-	-	-	-	-	-	-	-	0.1	-	-	-	RI, MS, ^{13}C-NMR
77	(*E*)-α-Bisabolene	1 531	1 761	-	-	-	-	-	-	-	-	-	0.7	-	-	-	-	-	-	-	-	-	-	-	-	-	RI, MS, ^{13}C-NMR
78	(10βH)-1β,8β-Oxido-cadina-4-ene	1 534	1 853	5.5	4.0	0.7	0.7	0.4	6.6	12.6	10.5	13.7	0.6	2.5	6.0	2.7	5.1	4.7	6.2	-	6.0	-	1.3	-	-	-	RI, MS, ^{13}C-NMR
79	β-Elemol	1 536	2 077	0.1	0.4	1.0	0.8	0.8	0.1	-	-	-	0.2	0.9	1.0	0.8	1.5	5.2	7.1	0.8	7.0	0.5	1.2	1.6	1.2	1.7	RI, MS, ^{13}C-NMR
80	(*E*)-Nerolidol	1 547	2 034	-	-	0.1	tr	tr	-	-	-	1.2	0.3	0.1	-	-	-	-	0.3	-	0.4	-	0.3	-	-	-	RI, MS, ^{13}C-NMR
81	Germacrene B#	1 549	1 818	2.4	3.6	12.2	15.7	15.6	2.4	1.6	2.5	1.4	17.6	13.2	1.3	15.9	1.0	4.2	0.6	2.6	1.6	4.5	0.1	3.5	4.1	2.5	RI, MS, ^{13}C-NMR

82	Palustrol	1 559	1 924	-	tr	0.1	-	-	-	0.6	0.7	0.5	-	-	0.1	-	tr	tr	0.1	0.1	tr	tr	-	0.1	-	-	RI, MS, ^{13}C-NMR
83	Santalenone	1 560	1 980	-	-	-	tr	-	-	7.1	6.9	7.2	tr	-	-	-	-	-	-	-	-	-	-	-	-	-	RI, MS, ^{13}C-NMR
84	*cis*-Sesquisabinene hydrate	1 562	2 079	0.2	0.1	tr	tr	tr	0.3	2.2	1.4	2.3	tr	-	-	-	-	-	-	-	-	-	0.1	-	-	-	RI, MS, ^{13}C-NMR
85	Germacra-1(10),5-dien-4β-ol	1 564	2 047	tr	-	-	-	-	-	0.4	0.6	0.5	-	0.1	0.2	0.1	0.2	0.3	0.3	0.5	0.3	0.2	0.1	0.7	-	-	RI, MS, ^{13}C-NMR
86	Caryophyllene oxide	1 567	1 973	0.1	0.5	1.1	0.8	0.9	0.3	2.9	1.5	1.8	0.5	0.3	tr	0.2	tr	0.1	tr	0.1	0.1	0.2	-	0.1	0.1	0.1	RI, MS, ^{13}C-NMR
87	Guaiol	1 581	2 119	tr	tr	tr	0.2	tr	tr	0.2	tr	tr	0.2	tr	tr	tr	tr	tr	tr	tr	tr	tr	-	tr	tr	tr	RI, MS, ^{13}C-NMR
88	Germacrene-D-8-one	1 584	2 066	6.8	4.2	0.3	0.3	0.1	8.4	0.9	1.1	0.8	1.1	1.0	3.6	1.2	3.4	2.8	3.2	tr	3.1	tr	0.6	tr	tr	tr	RI, MS, ^{13}C-NMR
89	Ledol	1 591	2 024	-	-	-	tr	tr	0.1	0.2	0.6	0.4	-	-	-	-	-	tr	tr	0.7	tr	0.3	-	1.0	-	-	RI, MS, ^{13}C-NMR
90	Humulene oxide II	1 597	2 042	0.3	0.2	0.1	0.1	0.1	0.4	1.7	0.7	1.3	0.1	-	-	-	-	-	-	-	-	-	-	-	-	-	RI, MS, ^{13}C-NMR
91	*epi*-Cubenol	1 605	2 046	tr	tr	-	-	-	tr	tr	0.2	-	-	-	-	-	-	-	-	-	-	-	-	-	-	-	RI, MS, ^{13}C-NMR
92	Alismol	1 609	2 245	0.1	0.2	0.3	0.5	0.5	tr	0.7	0.8	0.2	0.5	-	-	-	-	-	-	-	-	-	-	-	-	-	RI, MS, ^{13}C-NMR
93	Eremoligenol	1 614	2 196	-	-	-	-	-	-	0.7	0.2	0.5	-	0.2	0.1	0.1	0.1	0.1	0.1	0.3	0.1	0.2	-	0.4	0.3	0.5	RI, MS, ^{13}C-NMR
94	Cubenol	1 619	2 064	-	-	-	-	-	-	-	-	-	-	-	-	-	-	-	-	-	-	-	tr	-	-	-	RI, MS, ^{13}C-NMR
95	γ-Eudesmol	1 620	2 172	1.0	0.6	0.6	0.9	0.9	1.2	tr	0.5	0.3	0.3	0.2	0.1	0.1	0.2	0.5	0.6	0.2	0.8	0.1	tr	0.3	0.3	0.4	RI, MS, ^{13}C-NMR
96	δ-Cadinol	1 626	2 174	0.1	tr	tr	0.1	0.1	0.1	0.2	0.4	0.2	tr	-	-	-	-	-	-	-	-	-	-	-	-	-	RI, MS
97	Muurola-4,10(14)-dien-8β-ol	1 629	2 186	2.3	1.3	0.1	0.2	0.1	2.8	2.6	2.1	3.0	0.4	1.8	2.2	1.3	2.1	2.4	3.0	tr	3.2	-	0.3	tr	-	-	RI, MS, ^{13}C-NMR
98	β-Eudesmol	1 635	2 225	-	-	-	-	0.1	-	0.4	0.5	0.8	-	tr	-	-	tr	0.4	0.4	9.2	0.6	6.0	-	17.6	13.3	22.5	RI, MS, ^{13}C-NMR
99	α-Cadinol	1 637	2 231	0.5	0.5	0.4	0.8	0.7	0.6	0.2	1.0	1.7	0.3	tr	0.3	0.2	0.3	0.4	0.6	-	0.6	-	tr	-	-	-	RI, MS, ^{13}C-NMR
100	α-Eudesmol	1 639	2 216	-	-	-	-	tr	-	0.5	0.8	0.9	-	-	-	-	-	0.3	0.4	5.6	0.5	3.6	-	10.6	7.4	12.4	RI, MS, ^{13}C-NMR
101	Tumerone-ar	1 642	2 252	-	-	-	-	-	-	0.4	0.6	0.8	-	0.2	-	-	-	0.1	tr	0.4	-	0.2	-	-	-	0.1	RI, MS, ^{13}C-NMR
102	β-Bisabolol	1 653	2 144	tr	tr	0.1	0.2	0.2	0.2	0.1	tr	0.1	-	-	-	-	-	-	-	-	-	-	tr	-	-	-	RI, MS, ^{13}C-NMR
103	(7βH)-Germacrene D-8α-ol*	1 657	2 355	5.8	2.0	0.2	0.2	tr	6.1	0.2	0.5	0.3	0.8	1.1	2.1	0.6	2.1	3.6	3.5	-	2.8	-	0.7	-	-	-	RI, MS, ^{13}C-NMR
104	(7βH)-Germacrene D-8β-ol*	1 657	2 355	1.9	1.1	tr	tr	tr	2.6	tr	tr	tr	tr	0.5	0.6	0.2	0.8	1.3	1.2	-	1.2	-	0.2	-	-	-	RI, MS, ^{13}C-NMR
105	α-Bisabolol	1 664	2 234	0.8	0.4	tr	tr	tr	1.2	0.1	1.1	1.5	0.1	-	-	-	-	-	-	-	-	-	0.1	-	-	-	RI, MS, ^{13}C-NMR
106	*epi*-α-Bisabolol	1 667	2 208	0.1	tr	tr	tr	tr	0.1	0.3	0.2	0.5	-	-	-	-	-	-	-	-	-	-	-	-	-	-	RI, MS, ^{13}C-NMR
107	Germacrone	1 671	2 219	-	-	-	-	-	-	-	-	-	1.2	-	-	-	-	-	-	-	-	-	-	-	-	-	RI, MS, ^{13}C-NMR
108	Cadina-1(10),4-dien-8α-ol	1 672	2 306	-	-	-	-	0.1	0.1	0.9	0.7	0.9	0.1	0.2	0.1	0.1	tr	0.1	0.2	-	0.2	-	-	-	0.2	-	RI, MS, ^{13}C-NMR
109	Cadina-1(10),4-dien-8β-ol	1 676	2 276	5.5	2.6	0.5	0.6	0.2	6.9	1.3	1.2	1.9	0.9	3.2	3.9	2.2	4.0	4.8	5.8	-	6.2	-	0.3	-	-	-	RI, MS, ^{13}C-NMR
110	Cadina-4,10(14)-dien-8β-ol	1 678	2 280	0.3	0.1	0.3	0.8	0.7	0.7	0.1	1.0	1.4	-	-	-	-	-	-	0.2	-	0.1	-	-	-	-	-	RI, MS, ^{13}C-NMR
111	(1βH,5βH)-6,12-Oxido-guaia-6,10(14),11(12)-trien-4α-ol	1 754	2 519	0.1	0.3	0.8	1.1	1.1	0.1	2.6	2.9	0.9	0.3	0.3	-	0.1	-	0.1	-	0.1	-	0.1	-	0.1	0.1	0.1	RI, MS, ^{13}C-NMR
112	(6αH)-Germacra-1(10),4,7(11)-trien-6,12-γ-lactone	1 856	2 829	-	tr	0.1	0.1	0.2	-	0.8	0.9	-	tr	-	-	-	-	0.3	0.1	-	0.2	-	-	-	-	-	RI, MS, ^{13}C-NMR
113	(*E*)-Phytol	2 097	2 609	-	-	-	-	-	-	-	-	-	-	tr	-	-	-	-	-	tr	-	0.1	-	0.1	tr	0.1	RI, MS
	Hydrocarbon monoterpenes			20.3	32.0	30.4	19.9	23.5	11.2	6.0	6.5	3.9	25.6	14.0	23.7	21.1	24.2	19.4	19.5	26.7	18.1	23.3	70.9	21.4	23.9	15.7	
	Oxygenated monoterpenes			0.4	0.5	0.6	0.5	0.6	0.2	1.5	1.0	1.1	0.4	1.4	0.4	1.1	0.4	1.0	0.2	1.2	0.2	0.8	0.3	0.4	0.3	1.3	
	Hydrocarbon sesquiterpenes			41.4	40.9	45.4	51.5	53.1	41.0	45.6	46.6	44.2	61.2	53.2	46.3	54.7	47.0	41.0	38.0	49.4	39.3	57.8	22.2	37.1	42.8	40.9	
	Oxygenated Sesquiterpenes			34.1	23.3	19.6	23.7	19.1	41.3	42.8	41.7	47.7	10.5	22.2	21.0	16.2	20.5	29.7	33.7	19.0	33.9	13.8	5.5	34.3	24.7	39.8	
	Other compounds			0.0	0.0	0.1	0.1	0.1	0.0	0.1	0.3	0.1	0.1	0.0	0.0	0.0	0.0	0.0	0.0	0.0	0.0	0.8	0.0	0.1	0.0	0.5	
	**Total**			**96.2**	**96.7**	**96.1**	**95.7**	**96.4**	**93.7**	**96.0**	**96.1**	**97.0**	**97.8**	**90.8**	**91.4**	**93.1**	**92.1**	**91.1**	**91.4**	**96.3**	**91.5**	**96.5**	**98.9**	**93.3**	**91.7**	**98.2**	

Order of elution and percentages are given on an apolar column (BP-1), except components with an asterisk (*), where percentages are taken on a polar column (BP-20). (#) Thermolabile compound, percentage evaluated by a combination of GC-FID and ^{13}C-NMR data. RIa, RIp: retention indices measured on apolar and polar capillary column, respectively. tr: traces level (<0.05%). ^{13}C-NMR: compounds identified by NMR in the essential oil samples and obvious in at least one fraction of chromatography; *^{13}C-NMR (italic)*: compounds identified by NMR in fractions of chromatography.

Table S2. Plant material, essential oil extraction and climate data.

Samples	Leaves weight (g)	Essential oil weight (g)	Extraction yield (%)	Harvest site	Month	Monthly Mean Temperature (°C)	Season	Monthly Mean		Phenology
								Rainfall (mm)	Humidity (%)	
1	565.68	2.1796	0.385	Site 1	August 2016	25.8	Rainy	40-50	82	Shrub bearing neither flowers nor fruits
2	1301.67	4.1847	0.321	Site 1	August 2016	25.8	Rainy	40-50	82	Shrub bearing neither flowers nor fruits
3	1144.13	4.0351	0.353	Site 1	August 2016	25.8	Rainy	40-50	82	Shrub bearing neither flowers nor fruits
4	870.70	2.1854	0.251	Site 1	August 2016	25.8	Rainy	40-50	82	Shrub bearing neither flowers nor fruits
5	833.55	1.5173	0.182	Site 2	March 2016	30.7	Dry	0-5	56	Shrub bearing fruits
6	890.14	2.0424	0.229	Site 2	March 2016	30.7	Dry	0-5	56	Shrub bearing fruits
7	999.51	1.6992	0.170	Site 2	March 2016	30.7	Dry	0-5	56	Shrub bearing fruits
8	799.55	1.6901	0.211	Site 2	March 2016	30.7	Dry	0-5	56	Shrub bearing fruits
9	930.03	1.7502	0.188	Site 2	March 2016	30.7	Dry	0-5	56	Shrub bearing fruits
10	1302.01	1.5511	0.119	Site 3	January 2017	27.4	Dry	0	40	Shrub bearing neither flowers nor fruits
11	1432.22	1.5104	0.105	Site 3	January 2017	27.4	Dry	0	40	Shrub bearing neither flowers nor fruits
12	1159.18	1.8433	0.159	Site 3	January 2017	27.4	Dry	0	40	Shrub bearing neither flowers nor fruits
13	1102.01	1.2894	0.117	Site 3	January 2017	27.4	Dry	0	40	Shrub bearing neither flowers nor fruits
14	3108.37	4.8802	0.157	Site 4	March 2016	30.7	Dry	0-5	56	Shrub bearing neither flowers nor fruits
15	957.21	1.0146	0.106	Site 4	April 2016	28.7	Rainy	10-15	76	Shrub bearing neither flowers nor fruits
16	1191.52	1.6205	0.136	Site 4	March 2016	30.7	Dry	0-5	56	Shrub bearing neither flowers nor fruits
17	881.69	0.8729	0.099	Site 4	January 2017	27.4	Dry	0	40	Shrub bearing neither flowers nor fruits
18	752.99	1.0468	0.139	Site 4	March 2016	30.7	Dry	0-5	56	Shrub bearing neither flowers nor fruits
19	920.35	2.6323	0.286	Site 5	April 2016	28.7	Rainy	10-15	76	Shrub bearing neither flowers nor fruits
20	1031.85	2.6855	0.260	Site 5	April 2016	28.7	Rainy	10-15	76	Shrub bearing neither flowers nor fruits
21	1354.16	1.7510	0.129	Site 5	August 2016	25.8	Rainy	40-50	82	Shrub bearing neither flowers nor fruits
22	978.03	2.6760	0.274	Site 5	April 2016	28.7	Rainy	10-15	76	Shrub bearing neither flowers nor fruits
23	1130.33	1.0721	0.095	Site 5	August 2016	25.8	Rainy	40-50	82	Shrub bearing neither flowers nor fruits
24	1120.15	3.7642	0.336	Site 5	April 2016	28.7	Rainy	10-15	76	Shrub bearing neither flowers nor fruits
25	1035.19	3.0822	0.298	Site 6	April 2016	28.7	Rainy	10-15	76	Shrub bearing neither flowers nor fruits
26	1180.03	1.1332	0.096	Site 6	April 2016	28.7	Rainy	10-15	76	Shrub bearing fruits
27	1204.30	1.7339	0.144	Site 6	August 2016	25.8	Rainy	40-50	82	Shrub bearing neither flowers nor fruits
28	1152.96	2.4034	0.208	Site 6	August 2016	25.8	Rainy	40-50	82	Shrub bearing neither flowers nor fruits
29	965.47	1.3622	0.141	Site 6	August 2016	25.8	Rainy	40-50	82	Shrub bearing neither flowers nor fruits
30	914.65	2.7616	0.302	Site 6	April 2016	28.7	Rainy	10-15	76	Shrub bearing neither flowers nor fruits
31	1086.39	1.7653	0.162	Site 6	April 2016	28.7	Rainy	10-15	76	Shrub bearing flowers
32	999.23	2.6225	0.262	Site 6	April 2016	28.7	Rainy	10-15	76	Shrub bearing flowers
33	936.44	1.5385	0.164	Site 6	April 2016	28.7	Rainy	10-15	76	Shrub bearing flowers
34	1761.64	3.7523	0.213	Site 6	August 2016	25.8	Rainy	40-50	82	Shrub bearing neither flowers nor fruits
35	1030.2	3.9289	0.381	Site 1	August 2016	25.8	Rainy	40-50	82	Shrub bearing neither flowers nor fruits

36	1634.4	3.1168	0.191	Site 2	April 2016	28.7	Rainy	10-15	76	Shrub bearing fruits
37	1591.4	3.1113	0.196	Site 1	August 2016	25.8	Rainy	40-50	82	Shrub bearing neither flowers nor fruits
38	1828.5	3.9123	0.214	Site 2	April 2016	28.7	Rainy	10-15	76	Shrub bearing fruits
39	1051.6	4.0522	0.385	Site 5	April 2016	28.7	Rainy	10-15	76	Shrub bearing fruits
40	710.3	2.8717	0.404	Site 2	April 2016	28.7	Rainy	10-15	76	Shrub bearing fruits
41	435.5	1.6699	0.383	Site 7	July 2020	25.7	Rainy	60-75	89	Shrub bearing fruits
42	711.1	2.7034	0.380	Site 7	July 2020	25.7	Rainy	60-75	89	Shrub bearing fruits
43	396.6	1.5547	0.392	Site 7	July 2020	25.7	Rainy	60-75	89	Shrub bearing fruits
44	1454.5	4.2763	0.294	Site 8	February 2021	28.4	Dry	0-10	65	Shrub bearing flowers
45	450.9	1.5000	0.333	Site 8	February 2021	28.4	Dry	0-10	65	Shrub bearing flowers
46	452.9	1.7472	0.386	Site 8	February 2021	28.4	Dry	0-10	65	Shrub bearing flowers
47	784.2	3.9685	0.506	Site 8	February 2021	28.4	Dry	0-10	65	Shrub bearing flowers

Harvest sites locations: Bossématié forest, Region of Abengourou, Eastern Côte d'Ivoire, Site 1 (6°31′34.7″ N and 3°28′15.0″ W), Site 2 (6°29′26.0″ N and 3°29′11.7″ W), Site 3 (6°27′44.9″ N and 3°32′25.5″ W), Site 4 (6°25′44.5″ N and 3°32′40.7″ W), Site 5 (6°26′07.2″ N and 3°28′27.2″ W), Site 6 (6°23′43.4″ N and 3°25′59.4″ W). Haut-Sassandra forest, Western Côte d'Ivoire, Site 7 (6°53′40.2″ N and 6°55′36.3″ W), Site 8 (6°54′52.7″ N and 6°57′21.1″ W). Temperature, rainfall and humidity data (monthly mean) are from World AgroMeteorological Information Service (www.wamis.org).
